# Dr. Everly’s Case in the November Number

**Published:** 1887-01

**Authors:** 


### DR. EVERLY'S CASE IN THE NOVEMBER NUMBER.

Dr. Berridge writes:

"The nearest medicines to Dr. Everly's case are, *Baptis.*, *Cajuput.*, *Phos.*, *Psor.*, *Plat.*, *Sil.*, *Stram.*, which have 'delusion that he is in pieces,' or a similar sensation. Of these *Phos.*, *Plat.*, *Sil.* have aggravations of head symptoms from heat. *Phos.* (with *Bapt.* and Cajuput.) has 'delusion that he cannot get himself together.' I think *Phosphorus* is the *simillimum*, and should be glad to hear the result."

